# The Status and Future of Consciousness Research

**DOI:** 10.3389/fpsyg.2017.01719

**Published:** 2017-10-10

**Authors:** Morten Overgaard

**Affiliations:** Cognitive Neuroscience Research Unit (CNRU), CFIN, Institute of Clinical Medicine, Aarhus University, Aarhus, Denmark

**Keywords:** consciousness, consciousness disorders, measures of consciousness, neural correlates of consciousness, artificial consciousness

As papers about consciousness are so often introduced, consciousness was until few decades ago considered a philosophical problem only, and the current interest in empirical consciousness research was unforeseen. This development was of course influenced by the technological advancements in neuroscience during those decades, but more important and fundamental was a new openness to interdisciplinary integration of research questions, methods and arguments. Cognitive scientists and neuroscientists agreed that the philosophical problems of why and how there is consciousness are also their problems. Philosophers agreed that empirical evidence may resolve or at least influence this debate. Scientists across disciplines generally agree that consciousness is subjective, characterized by a kind of privileged first-person access.

Consciousness research has proven to be an actual and functioning discipline able to provide meaningful and reproducible results. Nevertheless, it has yet only scratched the surface in the attempt to solve some its bigger challenges, e.g., its many underlying questions of metaphysics (i.e., why does consciousness exist?) and questions of mechanisms (how does consciousness exist?).

One major obstacle for consciousness research is the lacking consensus of how to optimally measure consciousness empirically. Another major challenge is how to identify neural correlates of consciousness. This challenge clearly relates to the first as one needs to apply a measure of consciousness in order to identify its correlates. Current consciousness research is already occupied with these questions that may even be said to dominate the scientific debate.

As it will be argued below, consciousness research may face problems in the future that are currently less debated but which are logical extensions of the challenges above. It is a natural ambition when developing a measure of consciousness to be able to determine whether non-reporting subjects or even machines are conscious and of what. And it is a natural ambition when finding neural correlates of consciousness to understand how these correlates relate to a deeper metaphysical understanding of the relation between subjective experience and the physical substrate of the brain.

## How do we measure consciousness?

Historically, the attempt to “measure” consciousness has unfolded as a debate between direct and indirect approaches. Direct approaches, at least intuitively, are the most informative as participating experimental subjects here simply report about their own experiences. As subjective reports however have demonstrable limits (e.g., lack of insights into personal bias, memory problems etc.), many scientists have refrained from their use and insisted on the use of objective measures only (e.g., Nisbett and Wilson, 1977; Johansson et al., 2006).

Experiments on consciousness that are based on objective measures—the “indirect” approach—typically involve asking subjects to choose between alternatives, e.g., in forced-choice tasks. Although such methods may stay clear of classical limitations of subjective methods, they are confronted with other problems, which, according to some scientists, are greater. For one thing, objective measures must assume that the “threshold” of giving a correct response is the same as the “threshold” of having a subjective experience of the same content (Fu et al., 2008; Timmermans and Cleeremans, 2015). Furthermore, in order to arrive at any one particular objective method, one must have “calibrated it” to something else in order to know that this particular behavior can be considered a measure of consciousness—and not something else. This would typically involve associating a subjective report with a particular behavior—a process by which one would “import” all the weaknesses related to subjective reports that one tried to avoid in the first place (Overgaard, 2010).

Proponents of the “direct” approach have attempted to develop precise and sensitive scales to capture minor variations in subjective experience, e.g., the Perceptual Awareness Scale and gradual confidence ratings (Ramsøy and Overgaard, 2004; Sandberg and Overgaard, 2015). Although different approaches to this idea disagree about what consistutes the optimal measure (Dienes and Seth, 2010; Timmermans et al., 2010; Szczepanowski et al., 2013), they share the view that a detailed subjective report may be imprecise yet better than an indirect measure.

In recent years, the arsenal of indirect measures have been supplied with what is named “no-report paradigms.” Essentially, all paradigms using objective measures only are without report, so in a certain sense, paradigms labeled “no-report paradigms” have not introduced anything new. Nevertheless, experiments of this kind attempt first to associate a particular objective measure (e.g., a behavior or a brain activation) with conscious experience, and then to apply this measure as a measure of consciousness so that no direct report is needed (e.g., Frässle et al., 2014; Pitts et al., 2014). Such methods intuitively seem to circumvent some of the criticism mentioned above. However, and as mentioned above, the only way one may associate a phenomenon as nystagmus with conscious experience is by the direct use of introspection (to establish the “correlation”) (Overgaard and Fazekas, 2016).

It has been proposed that the best and most practical way forward is to combine methods and learn what we can from the results we get (Tsuchiya et al., 2016). Whereas this is most likely what is necessary, it is important to notice that different methods seem to generate different results, so that some methods are associated with the finding that the neural correlates of visual consciousness involve prefrontal activity, whereas other methods are associated with the finding that visual consciousness mainly involve occipital/parietal activity but not prefrontal.

## Neural correlates of consciousness

Most neuroscientific research on consciousness has had the explicit aim to identify the neural correlates of consciousness. Although it is rarely debated what we mean with a “neural correlate of consciousness,” most experiments aim to identify the minimal neural activations that are sufficient for a specific content of consciousness (Chalmers, 2000). Contrary to this, other scientists are preoccupied with finding neural correlates of consciousness “as such”—i.e., neural correlates that mark the difference between being dead, asleep, awake, etc., and which are not content-specific in the sense above.

With regards to the attempt to isolate neural correlates of conscious content, one central debate in recent years has been whether neural correlates of consciousness should primarily be associated with prefrontal cortex (“late” activations) or whether (visual) consciousness should be associated with occipital/parietal activations (“early” activations). According to most recent reviews and articles, evidence is lending support toward the latter view (Andersen et al., 2016; Koch et al., 2016; Hurme et al., 2017). According to this view, “late” activations are not actual correlates of consciousness, but are confounds associated with metacognition and report (e.g., Aru et al., 2012). Nevertheless, proponents of the opposite view—that consciousness is associated with prefrontal cortex activity—argue that “early” activations in fact represent preconscious states—i.e., information that is not yet conscious (e.g., Lau and Rosenthal, 2011).

According to other perspectives, this debate is partially misunderstood. Block (2005) argues that there may be two neural correlates of consciousness: One relating to phenomenal consciousness (the “early” activations), and one relating to access consciousness (the “late” activations). Others suggests that there is an identity between subjective experience and certain causal properties of physical systems rather than an identity between experience and particular brain parts (Tononi et al., 2016). According to the REF-CON model of consciousness, subjective experience is intrinsically related to a particular kind of “strategy” that makes information available for action (Overgaard and Mogensen, 2014; Mogensen and Overgaard, 2017). From this perspective, there need not be any “universal” correlate of consciousness at all. But even in such theoretical models according to which finding neural correlates of consciousness is very different from explaining consciousness, neural correlates of consciousness are essential as evidence to show how and if they work in practice.

There has been relatively more research into the neural correlates of the contents of consciousness than into “consciousness as such.” Research attempting to identify particular “levels” of consciousness obviously also face many methodological challenges, not least relating to contrastive analysis. Some studies have attempted to contrast healthy subjects with patients in vegetative state or minimally conscious state (Boly et al., 2013), although there are a number of problems: Some experiments indicate that not all such patients are unconscious (Owen et al., 2006), and—at the same time—most brain injured patients have many different lesions and, consequently, massive reorganization, which makes comparisons very difficult.

## The future challenges

The “upsurge” of interest in a science of consciousness did not begin but certainly took off with the publications of Chalmers (1995, 1996) and the Tucson-based conference series “Toward a Science of Consciousness”—soon to be further strengthened by the annual conferences organized by the ASSC (Association for the Scientific Study of Consciousness). Since then, much has happened in the attempt to discover neural and cognitive correlates of consciousness. It is however as uncertain today as it was then how exactly to apply these findings. In principle, there are many potential applications of consciousness research, but whereas some are extensions of the more fundamental questions (e.g., ethics and law), others are close to the heart of what consciousness research is (arguably) about, i.e., the mind-brain or mind-body problem.

One such fundamental problem relates to the fact that consciousness is subjective and in this way accessible from the first person only. Whereas we still have no universally accepted measures of consciousness, much progress has been made with regards to how one may grasp the content of an experience in the context of an experiment. One major future challenge will be how to measure consciousness “from the outside.” This problem is currently being faced in coma and vegetative state patients who either do not respond or respond in a minimal or strange fashion. It will very likely be an even greater challenge for a future science of consciousness to consider how to evaluate whether artificial systems (e.g., computers or robots) can be conscious or whether experience is a privilege for biological creatures. Essentially, these questions force us to try to make scientifically based decisions about how to measure conscious experience in highly different situations: In coma/vegetative state patients, there are little or no responses, yet a neural (however altered) system. In artificial systems, there may be high responsiveness (even, in principle, explicit expressions of being conscious) but no neural (biological) system.

One possibly even greater challenge will be to reintegrate the philosophical metaphysical debate into the scientific work. It will be a challenge to the future science of consciousness to demonstrate that empirical work on consciousness directly aids an understanding of the fundamental questions about consciousness. This challenge may seem unavoidable as the current preoccupation with cognitive functions and neural activations associated with subjective experience in most cases seems so directly linked to and motivated by the mind-brain problem. Existing data, however, seems to fit easily into every theoretical understanding of this problem. In and of itself, it seems not to be the case that evidence that perceptual experience is associated with—say—activity in primary visual cortex also provides evidence to determine whether consciousness should be seen as—say—identical to or metaphysically different from brain activity. Accordingly, it will require something “extra” to answer this challenge. Either, if possible, experimental investigations must be designed in order to “test” theoretical positions that currently are stated within the framework of philosophy of mind. Alternatively, experimental consciousness research must work even closer with theoretical consciousness research in order to make empirical data available as arguments.

## Future directions

The challenges highlighted above obviously only represent a few of the many scientific and theoretical issues that scientists in this area face. Consciousness remains one of the biggest scientific challenges among all disciplines as the most fundamental questions are not simply unanswered—it is still highly unclear how one should even begin to answer them.

Currently, consciousness research is often considered a “topic”—or even “niche”—under the umbrella of cognitive neuroscience. Nevertheless, consciousness researchers often point out that subjective experience is the underlying and fundamental reason for many questions in neuroscience. Scientists interested in the brain are often seeking answers to questions such as why we become addicted, how we remember, perceive, or solve problems. Such questions arguably presume conscious experience and make little sense without. Terms such as “memory” or “perception” do not solely refer to behavior, but also to particular kinds of conscious content which we know about from introspection. For this reason, one future ambition for consciousness research could be to become a more integral part of the overall ambition to understand the brain, and as such become part of the basic curriculum for any neuroscientist.

## Author contributions

The author confirms being the sole contributor of this work and approved it for publication.

### Conflict of interest statement

The author declares that the research was conducted in the absence of any commercial or financial relationships that could be construed as a potential conflict of interest.
